# Targeted Single Primer Enrichment Sequencing with Single End Duplex-UMI

**DOI:** 10.1038/s41598-019-41215-z

**Published:** 2019-03-18

**Authors:** Quan Peng, Chang Xu, Daniel Kim, Marcus Lewis, John DiCarlo, Yexun Wang

**Affiliations:** Research and Foundation Department, QIAGEN Sciences, Inc., Frederick, Maryland USA

## Abstract

For specific detection of somatic variants at very low levels, artifacts from the NGS workflow have to be eliminated. Various approaches using unique molecular identifiers (UMI) to analytically remove NGS artifacts have been described. Among them, Duplex-seq was shown to be highly effective, by leveraging the sequence complementarity of two DNA strands. However, all of the published Duplex-seq implementations so far required pair-end sequencing and in the case of combining duplex sequencing with target enrichment, lengthy hybridization enrichment was required. We developed a simple protocol, which enabled the retrieval of duplex UMI in multiplex PCR based enrichment and sequencing. Using this protocol and reference materials, we demonstrated the accurate detection of known SNVs at 0.1–0.2% allele fractions, aided by duplex UMI. We also observed that low level base substitution artifacts could be introduced when preparing *in vitro* DNA reference materials, which could limit their utility as a benchmarking tool for variant detection at very low levels. Our new targeted sequencing method offers the benefit of using duplex UMI to remove NGS artifacts in a much more simplified workflow than existing targeted duplex sequencing methods.

## Introduction

One hallmark of cancer development is the accumulation of somatic mutations in the body. In DNA from tumor biopsies and circulating cell free nucleic acids, mutations may be present in a very low fraction of all DNA molecules. Detection of these very low fraction mutations holds great promise in early diagnosis, monitoring the effectiveness of the targeted therapy and early intervention of drug resistant clones.

Recent advancement in next generation sequencing technology allows people to profile large genomic regions and look for somatic variations efficiently. However, detecting very low fraction of somatic mutations through NGS still presents significant challenges. Deep sequencing is required since many more wild type DNA molecules have to be sequenced before mutation bearing DNA can be observed due to its low fraction. The amount of sequencing depth depends on the detection limit one wishes to achieve. Because of the high sequencing cost associated with detecting low fraction mutations, most people adopted a targeted sequencing approach. Generally, targeted DNA enrichment before sequencing can be achieved through either specific probe hybridization capture^1^ or specific PCR primer amplification^2^. Probe capture based enrichment is often used for very large panels up to the whole exome, offers good coverage uniformity, but has a complex workflow and sometimes low enrichment specificity. PCR amplicon based enrichment has a short protocol and high enrichment specificity, especially for small panels. Although traditional amplicon enrichment using two locus-specific primers can severely limit the coverage of large indels and translocations, and is prone to SNP induced allele dropouts, newer single primer technologies based on ligation mediated PCR have alleviated those concerns^3–5^.

Given enough DNA input, an efficient enrichment process and enough sequencing depth, detecting mutations at very low fractions is still challenged by artifacts accumulated in many steps of the NGS workflow^6–8^. Those artifacts can arise from DNA base damage during the sample preparation, erroneous base incorporation by DNA polymerase during enrichment and library amplification, and errors from final sequencing readout. In order to better differentiate those NGS artifacts from real mutations at very low allele fractions, people have developed an error-correction mechanism through the incorporation of unique molecular identifiers (UMIs) in the NGS workflow^9–12^. In these protocols, UMIs (carried by short oligos) are attached to endogenous DNA fragments, carried along through enrichment and amplification, and sequenced together with genomic sequences. Amplification and sequencing artifacts can be removed from individual sequence read, based on the consensus of many reads bearing the same UMI (i.e. originating from the same molecule).

The caveat of the original UMI strategy is that any DNA base error present before UMI tagging or any polymerase error incorporated during the UMI tagging phase cannot be resolved. For example, it is well known that DNA base can undergo oxidative damage during storage, extraction, fragmentation and hybridization processes^6–8,13^. Although these very low level artifacts do not pose significant problems for most NGS applications, they can significantly impact our ability to detect real mutations below 0.2%, e.g. in circulating tumor DNA (ctDNA) applications. To further improve UMI’s error correction ability, a duplex UMI sequencing strategy (Duplex-seq) was first introduced by Schmitt *et al*.^14^. Duplex UMI sequencing further leverages the sequence complementarity of double stranded DNA to filter out false variants showing up in one but not the other strand of the original dsDNA fragment, and to gain further confidence of variants showing up in both strands of the original fragment. Recently, a number of studies have combined duplex UMI sequencing with hybridization capture enrichment to detect as low as 0.1% tumor derived cell free DNA with over 85–90% sensitivity and over 95% specificity^15,16^.

As mentioned earlier, probe hybridization capture tends to have low enrichment specificity for small regions. For example, in one study combining Duplex-Seq with one round of probe hybridization for a small region, only <5% reads mapped specifically to the target region^17^. Although by conducting two rounds of hybridization capture^17^ or using CRISPR/Cas9 enrichment^18^, read mapping specificity can increase to >90%, this brings in additional complexity to the library workflow. Therefore, a simple and specific enrichment method for duplex sequencing is desirable.

In our earlier work, we have reported a highly multiplex PCR enrichment system which integrates single-plex UMI and single primer extension (SPE) chemistry^5^. Although the system offers a simple workflow, high enrichment specificity/uniformity and good variant calling performance for as low as 0.5–1% allele fractions^19^, the accuracy for calling variants below 0.2% allele fraction is limited by the sample and workflow artifacts at similarly low levels. Changing from single-plex to duplex UMI would be desirable to combine the benefit of a simple enrichment workflow and increased variant calling accuracy.

**Figure 1:**
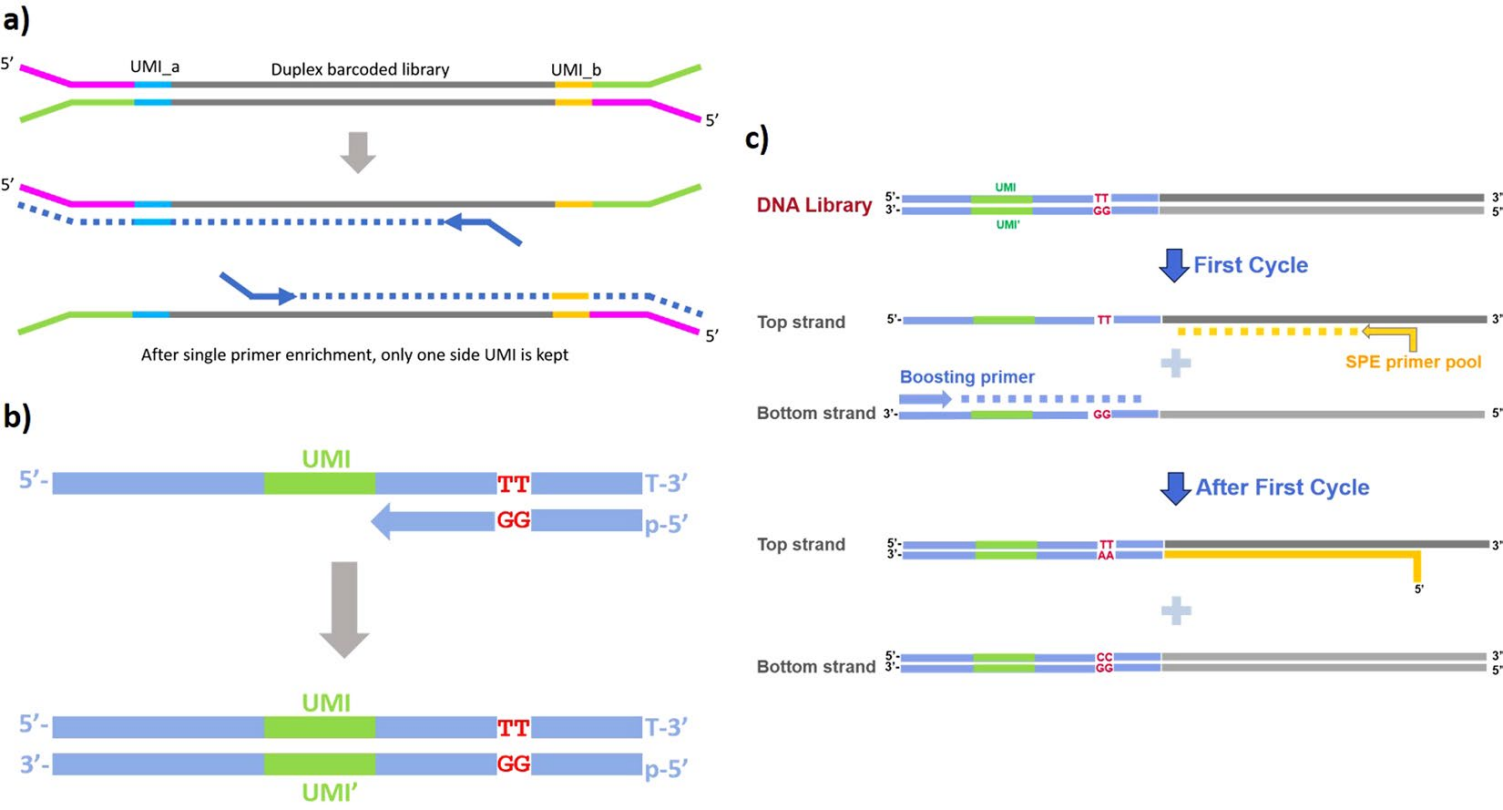
Design of single-end duplex-UMI adapter. (**a**) Schematics showing how previously described duplex UMI sequencing is not compatible with single primer PCR enrichment. The newly formed amplicons (dash lines) do not contain enough information to be grouped into corresponding duplexes. (**b**) Design and synthesis of the single end duplex-UMI adapter. Both UMI and strand barcodes (forming duplex UMI) are contained within a single adapter molecule and can be sequenced within one read. (**c**) Depiction of how duplex amplicons are encoded during the first few PCR enrichment cycles.

In the original Duplex-seq publication^14^, the duplex sequence information was assembled by tracking UMIs at both ends of a library fragment. Specifically, the chirality of a UMI pair from read1 and read2 sequences was used to decide whether two UMI pairs came from the same double stranded molecule. Thus pair-end sequencing was required. Probe hybridization capture of a Duplex-seq library preserves both UMI regions after the enrichment. If such duplex library is enriched by using single primer extension PCR, the resulting library fragment loses UMI at one end, which now contains the enrichment primer sequence instead of a UMI. Without UMI chirality, the original duplex relationship cannot be assembled (Fig. 1a).

In this paper, we described an improved system to enable duplex UMI sequencing together with highly multiplex PCR enrichment. We extended our smCounter2 statistical model to incorporate duplex UMI information into the variant calling process. We demonstrated our system’s performance for calling variants at 0.1–0.2% fractions, by using both commercial DNA references as well as in-house mixtures of “Genome in a Bottle” (GIAB) DNA references. Our data showed the successful implementation of our system and confirmed the superior performance of duplex UMI over single-plex UMI in multiplex amplicon sequencing. While using reference DNA from multiple sources we observed various degrees of artifacts around 0.1% fractions from the sample preparation process. Some DNA reference materials, depending on how they are prepared, may have very limited utility in defining the specificity of NGS assays for detecting variants at ~0.1% fractions across a wide target region.

## Results

### Design of the duplex UMI adapter for single primer enrichment workflow

To maintain the duplex molecule information without relying on paired-end UMI chirality, we redesigned the single end UMI adapter with additional barcodes for two strands. The strand barcode “TT” labels the original top strand and “GG” (read as “CC” during sequencing) labels the original bottom strand (Fig. 1b). The combination of a UMI and the strand barcode within a single read contains the full information as the original paired-end duplex-UMI. The modified duplex-UMI adapter can be easily made by annealing two synthetic oligos followed by extension (Fig. 1b). The strand barcodes “TT” and “GG” are chosen because among all barcode possibilities, the more stable G:T pseudo-base pairings introduce the least disruption to the oligo annealing and extension. We applied the single-end duplex-UMI adapter to our previously described SPE enrichment workflow (Fig. 1c). During the first cycle of the PCR enrichment, the original top stand with “TT” barcode is extended (i.e. captured) as the template by target-specific primers. At the same time, the original bottom strand with “GG” barcode is converted into a top strand sequence with “CC” barcode, which makes it available to serve as the template for specific primer enrichment in the next PCR cycles. As the result, both original top and bottom strands can be enriched by single primer extension and their duplex relationship is traceable by the combination of UMIs and strand barcodes.

### Low level base artifacts in *in vitro* prepared DNA reference materials

In order to evaluate the performance of our single-end duplex UMI adapter and to characterize both sensitivity and specificity of our duplex UMI-aware variant caller, we needed DNA samples with well characterized genome sequences and with some SNVs at <0.2% allele fractions. We made such reference materials by mixing one GIAB reference DNA (NA12878) into another (NA24385) at 0.4% or 0.2% ratios.

**Figure 2:**
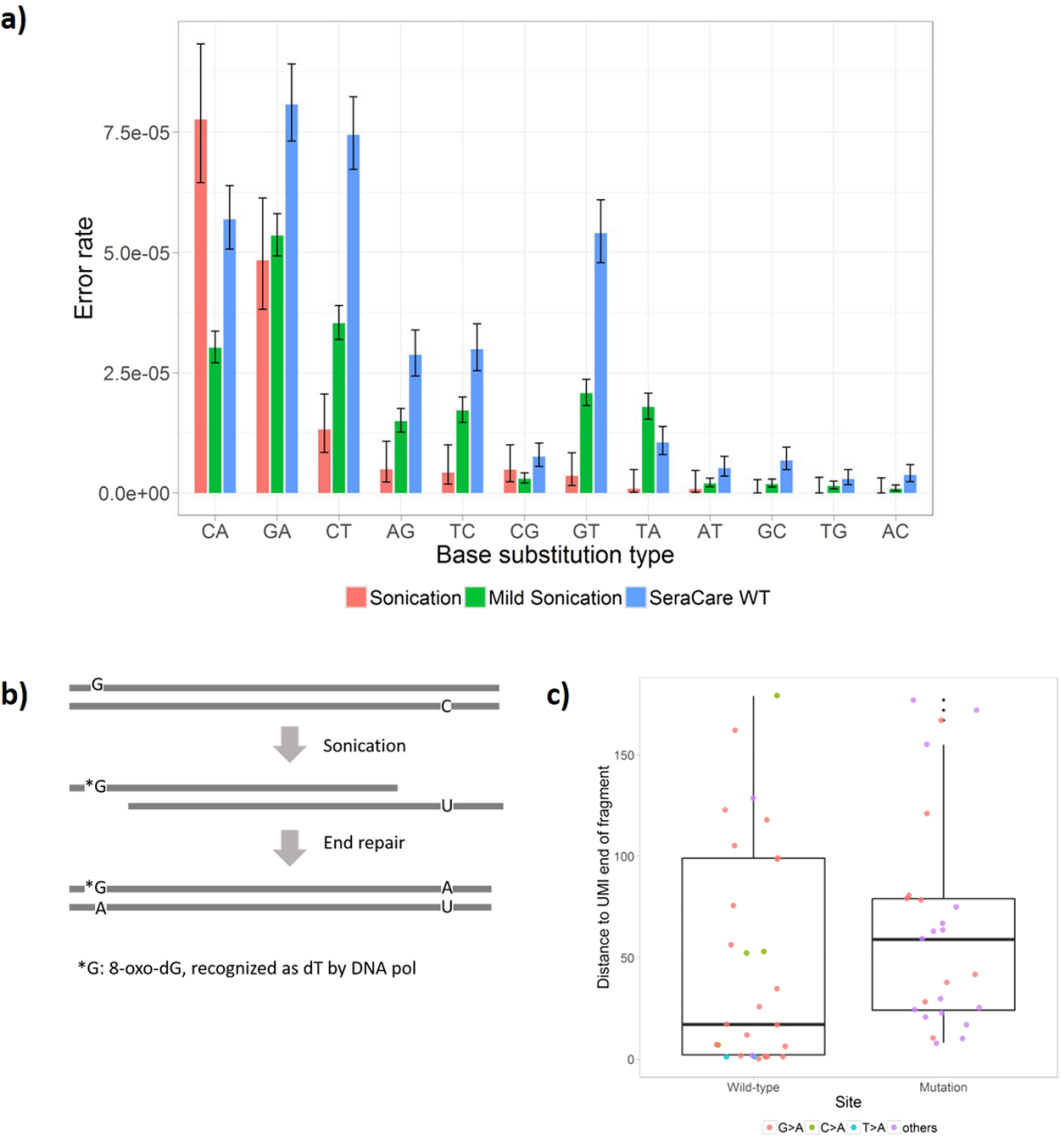
Analysis of base artifacts from sequencing results. (**a**) Observed panel-wise mean error rates from single UMI consensus reads for 12 different base substitution types from various DNA inputs. Here the base substitution type refers to the expected vs alternative nucleotide incorporated by DNA polymerase. For the same locus, the expected nucleotides to be incorporated will be different depending on which DNA strand is used as the template. The error bars indicate 95% confidence intervals of the error rates. The CIs were calculated using the Wilson’s method assuming Binomial distribution. (**b**,**c**) One possible mechanism (**b**) and evidence (**c**) for how artifacts were introduced during end repair process. We performed a two sample T-test to compare the mean of the two groups (25 real mutation sites and 29 false positives with duplex UMI support). The one-sided p-value is 0.12 under alpha level of 0.05.

While preparing and testing this reference material, we found that the DNA preparation steps before UMI tagging could greatly influence the apparent background base substitution rate calculated from UMI consensus reads. As shown in Fig. 2, we measured base substitution rates for all 12 types under different conditions. This rate was the sum of all errors introduced by fragmentation, end repair and first primer extension during enrichment PCR. When we used a typical sonication condition to fragment genomic DNA, there was a very high level of C > A substitution, compared to other types (Fig. 2a). This base substitution type was in concordance with the previously reported G-to-T transversion as the result of sonication induced oxidation of guanine^7,8^, which we reported as C-to-A (DNA polymerase incorporated a “A” instead of “C”). When a relatively mild sonication condition was applied to DNA, the observed C > A substitution dropped almost three folds (Fig. 2a). This result confirmed that sonication played a very important role in the background base errors observed in our sample. In theory, since the G-to-T transversion only affects one DNA strand at a given position, variant calling using duplex UMI should be able to distinguish real variants from damage induced G-to-T transversion. However, this strategy is limited by two observations in our experience. First, there were overwhelmingly more single-plex UMIs than duplex UMIs sequenced at each position, primarily due to the lower efficiency of sequencing duplex UMIs. We generally observed that duplex UMI represented ~25–40% of all UMIs sequenced (Table 1), which was consistent with an earlier report^15^. Because relying on duplex UMI alone will significantly limit the variant detection sensitivity, people have adopted a hybrid approach in using both single-plex and duplex UMI in variant calling^15^. Higher base artifacts in the sample and sequenced with only single-plex UMI can still limit the variant calling accuracy. Secondly, we observed that some of the base damage errors (G-to-T from dG oxidation or C-to-T from dC deamination) in one strand could be copied to the second strand during the end repair process, such that they would obtain duplex UMI support after adapter tagging (Fig. 2b). To assess this effect, we measured the distance between observed base substitution error and the 5′ end (the end where UMI is attached) of the DNA fragment. We hypothesized that the base substitution errors were more likely to happen near the end of DNA fragments if they were primarily introduced by end repair. In contrast, sonication damage or PCR amplification errors were more likely to be evenly distributed across the DNA fragments. As demonstrated in Fig. 2c, we observed strong tendency (one-sided p-value is 0.12, two sample T test) of those substitutions toward the end of DNA fragments, as measured by shorter distance to UMI region in the reads, compared to that of real SNVs. This result suggested that other than sonication damage, the majority of the remaining background noises from our in-house prepared DNA reference were likely introduced by the end repair process.

**Table 1:** Summary of the sequencing runs for various DNA reference materials.

Sample	0.2% DNA reference	0.1% DNA reference	Seraseq^®^ ctDNA Wild Type	Seraseq^®^ 0.25% ctDNA
Input amount	160 ng	160 ng	40 ng	40 ng
Number of enrichment primers	192	192	72	72
Size of target region	17,859 bp	17,859 bp	5,831 bp	5,831 bp
Total read pairs	14,428,738	23,761,224	3,469,754	4,535,556
Read pairs on-target	12,243,309	20,251,846	2,484,623	3,456,269
On-target rate	85%	85%	72%	76%
Mean read pair/primer	63,827	105,665	34,048	47,363
Mean UMI/primer	14,221	16,928	7,778	8,727
Mean Duplex UMI/primer	3,322	4,075	2,777	3,473
Mean read/UMI	4.5	6.2	4.4	5.5
% primer ≥0.2X mean read depth	99.5%	99.5%	98.6%	98.6%

To minimize artificially introduced SNV errors as mentioned above and be confident about the true specificity of our duplex UMI aware variant calling, we adopted a mild sonication condition and size-selected the fragments around 170 bp to create our in-house DNA references from the GIAB sample mixtures.

### Performance from in-house GIAB reference mixtures

Using the in-house DNA references prepared from mild sonication, we evaluated the performance of our duplex UMI enabled single primer enrichment workflow, by performing targeted sequencing with the 192 plex panel. The sequencing results are summarized in Table 1. We obtained 14,221 and 16,928 average UMI coverage depth for 0.2% and 0.1% DNA reference respectively. The ratios between duplex UMI and total UMI recovered were roughly 23~24%. More than 85% of the reads were useable and on target in both datasets. The coverage uniformity was also high, with greater than 99% of target regions with at least 20% of the mean read depth.

**Figure 3:**
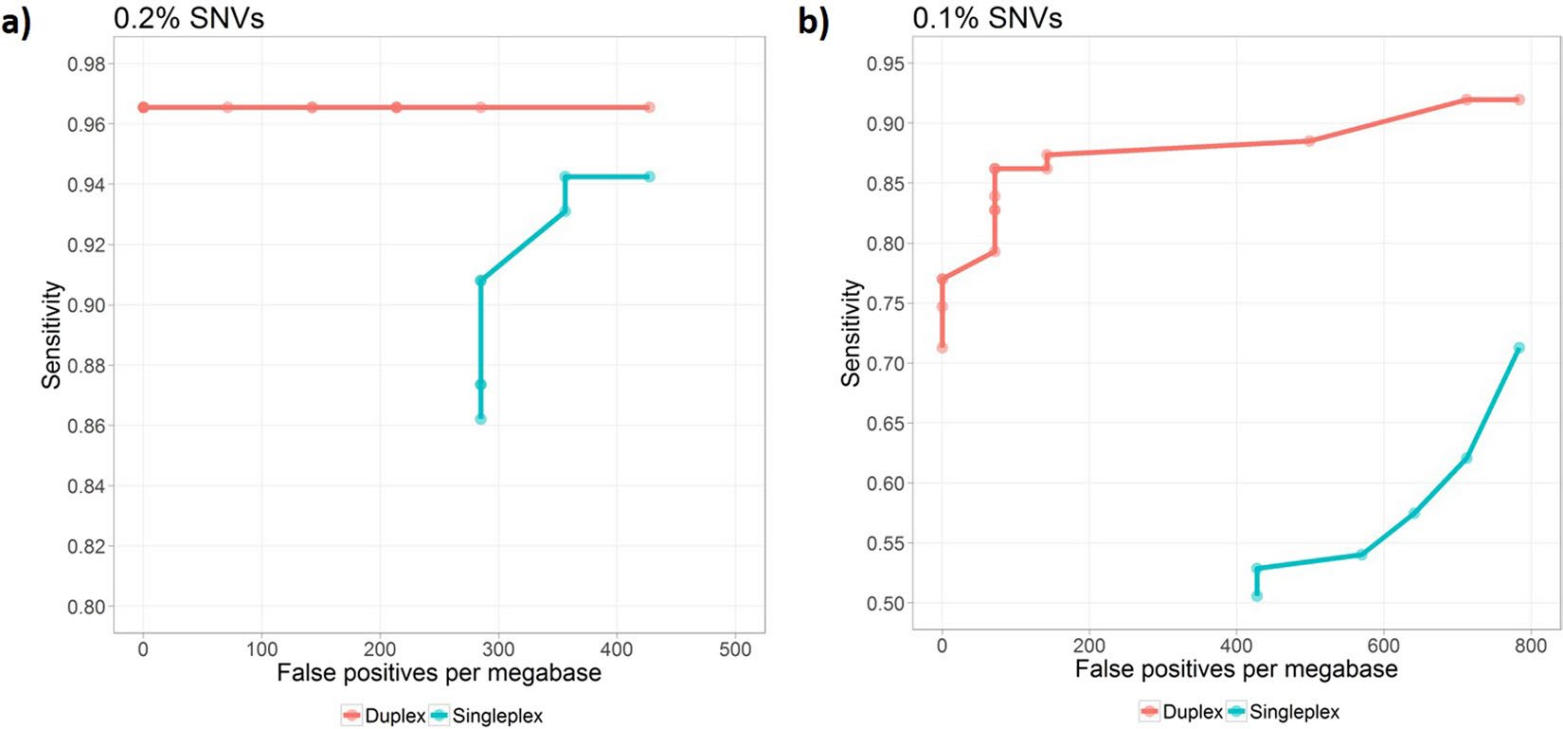
Sensitivity and specificity for detecting 0.1% and 0.2% SNVs from in-house made DNA reference. ROC curves for 0.1% SNVs (**a**) and 0.2% SNVs (**b**). “Duplex” represent variant calling performance using both single-plex and duplex UMI information. “Single-plex” represent variant calling performance by treating all UMIs as single-plex.

We then optimized our variant calling pipeline using these high quality datasets and achieved 96.6% SNV sensitivity with zero false positive on 0.2% DNA reference (Fig. 3a). The performance on 0.1% DNA reference was lower, due to sample artifacts introduced even under mild sonication conditions. However, we were still able to achieve 77% sensitivity with zero false positive, and 86.2% sensitivity with only one false positive (Fig. 3b). One false positive in this panel is proportional to ~71 false positives per mega bases (Mbp) region. Because of relatively small target size, this generalization can have high variability.

Next, we set out to evaluate the impact of duplex UMIs on variant calling. Of all the UMIs obtained from each sample, typically 25–40% were duplex UMIs (Table 1). We did variant calling again with the same datasets, but treating all UMIs as single-plex. Without the duplex UMI leverage, we achieved 62% sensitivity with 10 false positives for 0.1% variants and 95% sensitivity and 8 false positives for 0.2% variants (Fig. 3a,b). This result confirmed that duplex UMI could greatly reduce false positives, by utilizing complementary information from both strands of the same dsDNA.

### Performance from commercial NGS reference materials

To extend the scope of our evaluation, we tested our protocol using a commercially available NGS reference material with verified low fraction SNVs and well characterized genomic background. We chose to use Seraseq^®^ ctDNA v2 reference and designed a new 72 plex panel targeting 38 clinically-relevant mutations (25 SNVs and 13 indels), all at the same specified allele frequency in this reference material. As shown in Table 1, with 40 ng Seraseq^®^ ctDNA input, we were able to get ~8,000 average UMI coverage depth for both wildtype and 0.25% mutation mix at medium read per UMI sequencing depth. This level of UMI recovery was much higher than what we could achieve using our in-house prepared DNA reference materials. The ratios between duplex UMI and total UMI recovered were also higher at 35~39%.

**Figure 4:**
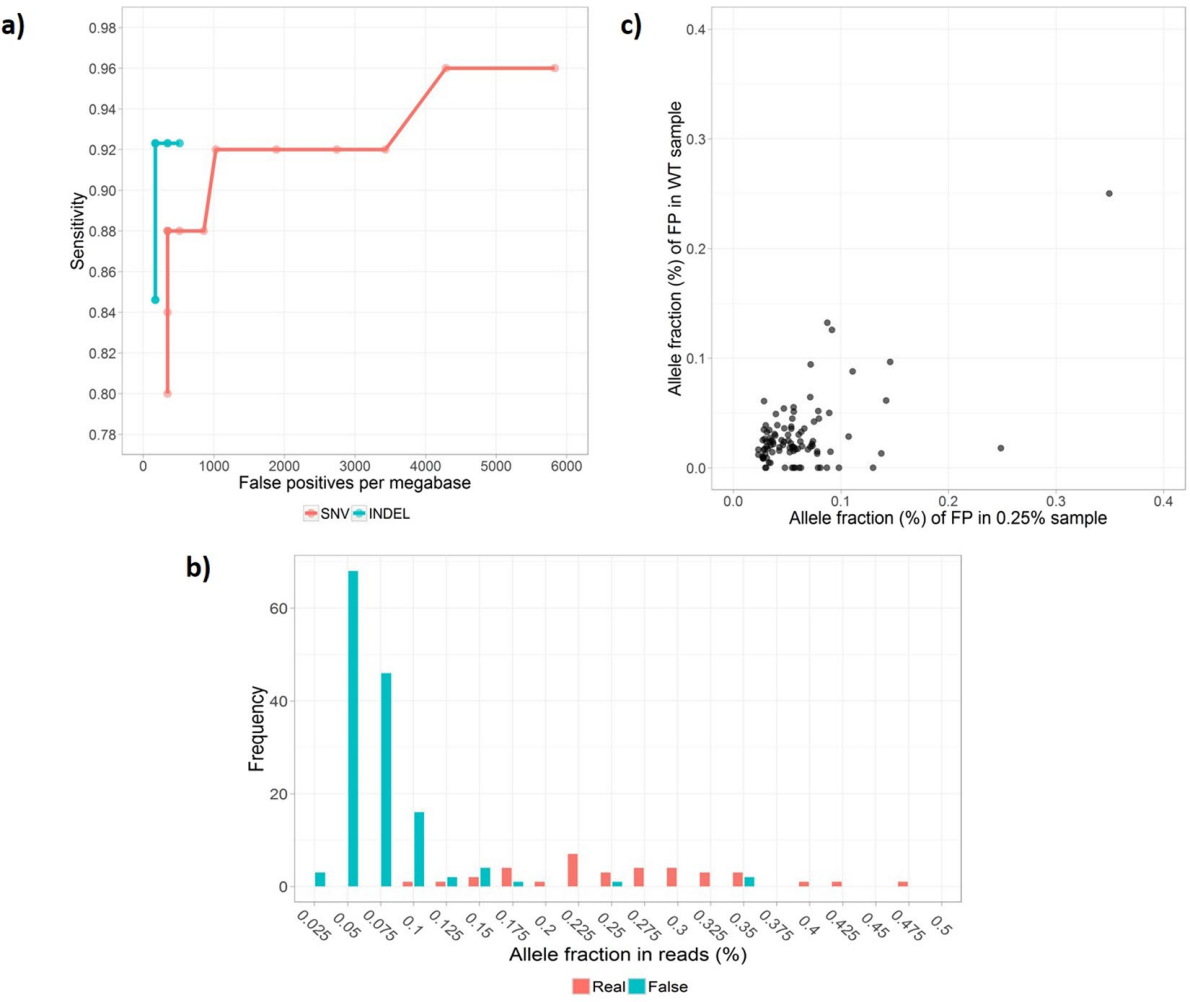
Detecting 0.25% variants from commercial NGS reference. (**a**) Sensitivity and specificity for detecting 0.25% SNVs and indels from Seraseq^®^ ctDNA mix. For the small target region, one false positive is proportional to 171 false positives/Mbp region. (**b**) Distribution of allele fractions for all variants seen in the Seraseq^®^ 0.25% cfDNA mix. Variants were identified from UMI consensus reads and no variant calling threshold was applied. Most of the false variants were below 0.1% allele fractions and were well separated from the real variants. (**c**) Around 80% of the low level false variants observed in Seraseq^®^ 0.25% ctDNA mix were also seen at similar fractions in the corresponding wildtype control. Since the 0.25% mix was made using wildtype control as the background, this strongly suggested that those false variants were intrinsic artifacts in the sample but not random errors from the enrichment and sequencing.

We applied the same duplex UMI-aware variant caller to this dataset from Seraseq^®^ 0.25% ctDNA mutation mix, and found that the overall SNVs calling performance was inferior to our in-house 0.2% DNA reference data (Fig. 4a). More false positives were called at the comparable detection sensitivities. For example, 2 false SNVs were called when SNV detection sensitivity was at 88% and 1 false indel was called when indel detection sensitivity was at 92%. Due to the relatively small target region, 1 false positive is proportional to 171 false positives/Mbp. More than 90% of the false positives were at allele fractions around or below 0.1% (Fig. 4b).

All of the false positives we identified in Seraseq^®^ 0.25% ctDNA mutation mix were supported by duplex UMIs. This led us to speculate that most of them could be artifacts generated during the production of this ctDNA mix, similar to what we observed in our in-house DNA reference. This hypothesis is supported by the following observations. First, according to the vendor, the 0.25% ctDNA mutation mix was made by titration using wild-type control as the background. When we sequenced the Seraseq^®^ ctDNA wild type control and calculated the base substitution errors, to our surprise, we found that the error rates were much higher than those from our in-house DNA reference prepared under mild sonication (Fig. 2a). The base substitution profile also shared great similarity to those from our sonication condition. Secondly, the same false positive SNVs were observed from both wild-type and 0.25% ctDNA mutation mix (Fig. 4c) as well as from independent enrichment sequencing experiments. This strongly suggests that those observed false positive SNVs were likely pre-existing artifacts in the sample at very low levels. Based on these and personal communications with the SeraCare, we believed that artificial base damages were also introduced during the fragmentation and preparation of this Seraseq^®^ NGS reference material. Because those artifacts were present at around or below 0.1% fractions, this Seraseq^®^ NGS reference material couldn’t be used to accurately define variant calling specificity for SNVs at around 0.1% allele fractions across a large region.

## Discussion

In recent years, duplex sequencing has become a leading high throughput method for detecting very low fraction variants from liquid biopsy. By leveraging the complementarity of individual dsDNA molecules, duplex sequencing has the superb ability in distinguishing real variants from various artifacts in the sample as well as introduced during the NGS workflow. Here, we successfully implemented a novel approach to enable duplex sequencing after highly multiplex PCR enrichment, through a unique duplex UMI adapter design. Comparing to existing approaches, our duplex information is encoded separately from UMI and does not depend on the chirality of two UMI regions. In principle, our single-end duplex-UMI adapter design can also enable duplex sequencing on a single end sequencing platform. Using two panels and two sources of NGS reference materials as examples, we demonstrated high enrichment specificity and uniformity of our targeted duplex sequencing protocol for small genomic regions. The amount of duplex UMI molecules recovered relative to the input DNA and the relative abundance of duplex UMI vs. total UMI after sequencing were in line with other duplex sequencing results. For example, from Supplementary Fig. 3b of Newman *et al*. paper, we estimated that ~4000 total hGEs (haploid genome equivalents) and ~800 duplex hGEs could be observed from 32 ng normal cfDNA input (or ~10,000 hGEs) after their duplex sequencing^15^. Our enriched sequencing library can be prepared within a day (8 hours). In comparison, standard duplex sequencing library or CRISPR-DS library with hybrid capture enrichment would require 2–3 days and more steps to finish^18^. We showed that the additional duplex UMI information greatly improved variant calling accuracy for mutations present at 0.1–0.2% fractions, primarily by reducing low level false positives. This confirmed the power of duplex UMI to suppress sequencing errors as reported in earlier studies. Additional improvement in variant calling sensitivity and specificity may be achievable by fine tuning the statistical model around duplex UMI. By performing single primer PCR enrichment together with duplex sequencing, we combined the benefits of PCR based enrichment (workflow simplicity, short turnaround time, enrichment specificity) and the high accuracy of duplex sequencing for the first time.

While evaluating the variant calling performance of our system, we observed significant number of background artifacts in both our in-house sonicated DNA reference and commercial NGS reference materials, which affected our variant calling specificity at 0.1% allele fractions. We have evidence to suggest that most of these artifacts at very low levels were base damages introduced during acoustic shearing of genomic DNA when creating in-house and commercial reference materials. For naturally occurring cfDNAs from liquid biopsy, such base damage artifacts should be at a much lower level, because no additional DNA fragmentation or extensive end repair is needed for preparing real cfDNA libraries. We believe our targeted duplex sequencing method can achieve even higher specificity with naturally occurring cfDNA samples than with *in vitro* prepared DNA reference materials.

During the course of this work, we found that the lack of good cfDNA reference was one of the limiting factors to accurately benchmark the variant detection performance of cfDNA related NGS methods. In an ideal case, the reference materials should mimic the size distribution and fragment end property of naturally occurring cfDNA. The sequence of reference materials needs to be well characterized with a set of known variants at various and relatively low allele fractions. The materials should also be free of low fraction artifacts. Fragmentation by sonicating purified genomic DNA introduces background base damages, thus limiting the assessment of real variant detection specificity. In addition, the end property of sonicated DNA is different from naturally occurring cfDNA. Sonicated gDNA, such as our in house DNA reference, has lower adapter ligation efficiency, which can lead to lower UMI molecules and reduced variant calling sensitivity than real cfDNA with the same amount of inputs. Certain commercial NGS reference materials, such as Seraseq^®^ ctDNA reference, may better resemble the size distribution and end property of naturally occurring cfDNA, so that we see better library yield than sonicated gDNA. However, their variant profiles are usually limited to a few hot spots, and other background regions are not well characterized. In addition, low level artifacts not naturally in the sample may be introduced during the preparation of the reference materials. For these reasons, most existing cfDNA reference materials are good for evaluating variant detection sensitivity, but inadequate for evaluating variant detection specificity across a large region. At the time of this work, we were not aware of any cfDNA reference materials in the field which met all the requirements.

Because the variant calling performance demonstrated by our duplex sequencing protocol was based on testing *in vitro* made reference materials, which do not fully resemble naturally occurring ctDNA or cfDNA, additional considerations should be noted when trying to extrapolate our results to ctDNA detection from real cell-free DNA. First, in addition to the above mentioned differences in base damage and library efficiency, real cell-free DNA fragments do not have fully random end positions^20^. For example, ctDNAs deriving from various tumor origins have different end positions from normal plasma cfDNA^20,21^, which is believed to derive primarily from normal blood cells^22^. The difference in cfDNA and ctDNA end positions can be leveraged to boost ctDNA detection in real cfDNA^20,21^, which is not possible with *in vitro* made reference materials. Secondly, the sensitivity in detecting individual low level variant, as shown in our study, should not be equated to the sensitivity in ctDNA detection, which is influenced by additional factors, such as the amount of cfDNA (e.g. ~1–50 ng/ml in healthy plasma vs. >100 ng/ml in cancer patients^23^) and the actual ctDNA abundance (e.g. 0.1–23% in one study^24^). Due to lower adapter ligation efficiency from sonicated DNA, we had to use much more *in vitro* reference materials in order to observe 0.1–0.2% variants in our NGS data. Although we believe cfDNA will have a much higher library efficiency, the actual amount of UMI captured using our protocols needs to be further studied using real cfDNA. The higher number of cfDNA molecules captured will be the foundation for higher sensitivity in ctDNA detection. Additionally, the sensitivity in detecting ctDNA could be higher than the sensitivity in detecting individual variant. Newman *et al*. demonstrated in their study that, by relying on an ensemble of variants for ctDNA detection, higher sensitivity could be achieved^15^. Because of these considerations, the real sensitivity in ctDNA detection achievable by our method will need to be further evaluated using real cfDNA from patients.

## Methods

### Preparation of *in vitro* GIAB sample mixtures

Human genomic DNA samples of NA12878 and NA24385 were purchased from Coriell Institute. Sample mixtures were created based on the actual amplifiable DNA in each sample, resulting in 0.2% of NA12878 DNA mixed in the NA24385 DNA. The resulting DNA mixture contains NA12878 variants present at 0.1–0.2% fraction. Homozygous SNVs unique to NA12878 are at 0.2% in the mixture, while heterozygous SNVs are at 0.1%. The 0.4% mixture was prepared in a similar fashion, resulting in 0.4% homozygous SNVs and 0.2% heterozygous SNVs.

To make the size distribution similar to cell-free DNA in plasma, we fragmented 1 μg genomic DNA mixtures in 30 μL water using Bioruptor 300 (Diagenode), followed by 1.0X/1.0X double size selection using AMPure XP beads (Beckman Coulter). The average size of the recovered DNA was measured around 170 bp by Agilent Bioanalyzer and its concentration was determined by Qubit dsDNA HS Assay Kit (Thermo Fisher Scientific). The size selected 0.4% mixture and 0.2% mixture were referred as 0.2% DNA reference and 0.1% DNA reference respectively. Normal sonication settings were 30 cycles of 30 seconds on and 30 seconds off with low power. Mild sonication settings were 3 cycles of 30 seconds on and 30 seconds off with low power.

### Custom panel design

The first panel was designed to target randomly selected 87 coding SNPs present in NA12878 but not in NA24385 (Supplementary Information). Here, we chose to work with a small region so that a deep sequencing run can be achieved within the MiSeq capacity. In total, 192 primers (“192 plex panel”) were designed using custom scripts, balancing coverage, primer Tm, dimer potentials and predicted specificity within human genome (Supplementary Information). For variant calling purpose, the total targeted region in the 192 plex panel was 17,859 bp. The second panel was designed to target specific SNVs and indels present in Seraseq^®^ ctDNA v2 reference materials (SeraCare). Different from SNPs in the GIAB reference, SNVs and indels in Seraseq^®^ ctDNA reference were all cancer related. A total of 72 primers (“72 plex panel”) were selected so that assumed 150 bp amplicons from those primers can cover all 38 SNV and indel loci (Supplementary Information). The total targeted region for variant calling in this 72 plex panel was 5,831 bp. A 5′ universal sequence (AATGTACAGTATTGCGTTTTG) was added to all primer sequences and all oligos were synthesized by IDT.

### Preparation of duplex adapter

The duplex adapter was produced by primer annealing and extension reaction. Briefly, in 1X NEBuffer 2 (New England Biolabs) oligonucleotide ds_IL_Adapter_1 (5′/5InvddT//iisodG//iisodG/CCTACACGACGCTCTTCCGATCTNNNNNNNNNNNNTTCTGAGCGATTATAGGAGTCC*T3′) at the final concentration of 50 μM was mixed with oligonucleotide ds_IL_Adapter_2 (/5phos/GGACTCCTATGGTCGCTCAGAA) at the final concentration of 100 μM. The oligo mixture was denatured at 95 °C for 5 min, and then cooled down to 25 °C at a rate of 1 °C/min. The annealed complex was purified using MinElute PCR Purification Kit (QIAGEN), and eluted in 11.5 μL EB. 10 μL of the elution were added into the extension reaction with the final concentrations of 1X NEBuffer 2, 0.5 mM each dNTPs, and 0.0625 U/μL DNA Polymerase I (New England Biolabs). The reaction was incubated at 25 °C for 30 mins and held at 4 °C before cleanup using MinElute PCR Purification Kit. The purified duplex adapters were checked by agarose gel electrophoresis for size and purity.

### Library and enrichment protocol

DNA end repair, A-addition, and adapter ligation were performed with variable amounts of DNA by using GeneRead DNA Library I Core Kit (QIAGEN), except that GeneRead adapter was replaced by 0.2 or 0.5 µM duplex adapter depending on the DNA input amount. To ensure complete removal of free barcoded adapters and adapter dimers after ligation, each reaction was purified for two rounds using 1.2X AMPure beads and eluted into 18 μL water. The entire purified library was then mixed on ice with 20 nM each target primer, 400 nM IL2 primer (AATGATACGGCGACCACCGAGATCTACACTCTTTCCCTACACGACGCTCTTCCGATCT), 1X VeraSeq buffer II, 0.65 mM each of dNTP, 0.5 mM MgCl_2_, 0.5X Q-solution (QIAGEN) and 0.5 μL VeraSeq2.0 High-Fidelity DNA polymerase (Enzymatics) in a total volume of 40 μL. The PCR enrichment condition was: 95 °C for 3 min; 8 cycles of 98 °C for 20 sec and 65 °C for 10 min; 72 °C for 5 min. Enriched library was purified for two rounds using 0.9X AMPure beads and eluted in 22 μL water. Purified library was combined with 400 nM IL-Forward primer (AATGATACGGCGACCACCGA), 400 nM IL-Index primer (CAAGCAGAAGACGGCATACGAGATCGTGATGTGACTGGAGTTCAGACGTGTGCTCTTCCGATCTAATGTACAGTATTGCGTTTTG, index region underlined. Index A001 for Illumina TruSeq HT is shown here as an example), 1X UPCR buffer (QIAseqTargeted DNA Panel, QIAGEN) and 1 μL HotStarTaq DNA polymerase (QIAGEN) in a volume of 20 μL. The universal PCR condition was: 95 °C for 13 min, 98 °C for 2 min; 20 cycles of 98 °C for 15 seconds and 60 °C for 2 min; 72 °C for 5 min. The DNA library was purified once using 0.9X AMPure beads and eluted in water. Final libraries were quantified by Agilent 2100 Bioanalyzer and sequenced on Illumina MiSeq (pair-end, 2 × 150 bp) following manufacturer’s user manual (Illumina).

### Read processing

The reads were processed in four steps. First, exogenous sequences including UMI, strand label, and PCR and sequencing adapters were identified and removed. The UMI and strand label were temporarily appended to the read identifier for downstream analysis. Second, the trimmed reads were mapped to the reference genome using BWA-MEM. To reduce ambiguous mapping, reads with very short endogenous sequence or low mapping quality were removed. Gene-specific primers were soft-clipped during mapping. Third, to account for sequencing and PCR errors in the UMI sequence, a UMI family with much smaller read count was merged with a much larger read family if their UMIs were within one edit distance and their random fragmentation sites were within 5 bp. Fourth, the UMI and strand label were removed from the read identifier and saved as custom tags in the aligned reads (BAM format).

### Estimation of the panel-wise mean base substitution error rates

We focused on the base substitution errors that occurred before or during UMI tagging phase or the first cycle PCR reaction, because such errors couldn’t be resolved by the single-plex UMIs and therefore determined our assay’s limit of detection. We used cfDNA mimics from the GIAB sample mixtures (Table 1) to estimate the mean base error rate for each type of substitution (Fig. 2a). The estimation was restricted to the GIAB high confidence region within the 192 plex panel, after removing the known mutational (from either sample) sites.

At each site, reads with the common UMI and strand label were grouped together as a UMI family. If at least 80% of the reads in the UMI family had the same allele, and at least 80% of the high-quality reads (defined by a set of thresholds on base quality, mapping quality, number of mismatches from the reference genome, and coverage of the homopolymer region) agreed with that allele, the UMI family was said to have a consensus. UMI families that failed to reach a consensus were removed because errors with only partial UMI support were more likely to have happened during the late-cycle PCR reaction or sequencing. Singleton UMIs (UMIs with only one read fragment) were also removed because errors supported by a single read fragment would likely be a sequencing artifact due to relatively high sequencing error rate. We assumed that for the remaining UMI families, a non-reference consensus was the result of a base substitution error that occurred before or during UMI tagging phase or the first cycle PCR reaction. Under this assumption, we could estimate the panel-wise mean background error rates as follows. We assume that at site *i* with the reference base *R*_*i*_ = *A*, there are *N*_*i*_ qualified UMIs and *n*_*i*_ of them have the consensus allele of G (*n*_*i*_ could be zero). The panel-wise mean A > G error rate can be computed as $\frac{\sum _{i}I({R}_{i}=A){n}_{i}}{\sum _{i}I({R}_{i}=A){N}_{i}}$, where $I({R}_{i}=A)$ is an indicator that takes value 1 if the reference base is A and 0 otherwise. We computed the panel-wise mean error rate for each type of substitution and illustrated them in Fig. 2a.

### Estimation of the distribution of base substitution error rates

Although the mean error rates provide enough information to guide the assay development, they are inadequate for variant calling under the smCounter2 framework^19^, where a probability distribution (rather than the mean) of the error rate is required. Unfortunately, a distribution could not be estimated using these two datasets due to inadequate UMI coverage. Under mild sonication, the highest base substitution error rate (G > A) is about 5 × 10^−5^ (Fig. 2a), whereas the mean UMI coverage of these datasets are below 20,000. Therefore, zero non-reference UMIs were observed at most of the wild-type sites. To get enough UMI coverage, we combined the reads from 10 independent Duplex-seq libraries on a 10-primer panel. The combined dataset accumulated over 100,000 non-singleton UMI per site. We used both UMI and sample index to separate DNA molecules to avoid barcode collision (two DNA molecules tagged with the same UMI).

For this analysis, we computed the error rates at each site instead of aggregating across the panel. We used a Beta distribution to model the error rate distribution (Supplementary Fig. 1). The Beta distribution parameters were estimated using the maximum likelihood method with R package *fitdistrplus*^25^. Unfortunately, the UMI depth of this dataset was still not enough for certain base substitution types whose error rates were at the magnitude of one per a million. Therefore, we only estimated the distribution of the G > A error rate. For variant calling, we conservatively assumed that all base substitutions had the same error profile as G > A.

### Variant calling with duplex UMI

We treated the variant calling as a model selection problem. The null model (*M*_0_) assumes that there is no mutation, therefore all non-reference UMIs are caused by background errors. The alternative model (*M*_1_) assumes that all non-reference UMIs come from real mutations. Suppose that at a specific site, after removing singleton and non-consensus UMIs, there are *N*_*1*_ single-plex and *N*_2_ duplex UMIs in total and among them, *n*_1_ single-plex and *n*_2_ duplex UMIs have the non-reference allele. We used the log likelihood ratio $\mathrm{log}\,\frac{P({n}_{1},\,\,{n}_{2}|{N}_{1},\,\,{N}_{2},\,{M}_{1})}{P({n}_{1},\,\,{n}_{2}|{N}_{1},\,\,{N}_{2,}{M}_{0})}$ to measure the evidence in favor of *M*_1_. The calculation of the log likelihood ratio is given in the Supplementary Information.

## Supplementary information

Additional information on error rate distribution and variant calling model

List of target variants and enrichment primers

## Data Availability

The datasets generated in the current study are available from the corresponding author upon reasonable request.
