# Supplementary material for: Targeted Single Primer Enrichment Sequencing with Single End Duplex-UMI
Source: Sci Rep. 2019 Mar 18;9:4810. doi: 10.1038/s41598-019-41215-z (PMC6423013; doi:10.1038/s41598-019-41215-z)
Supplement: Supplementary file 1 — Additional information on error rate distribution and variant calling model [file 41598_2019_41215_MOESM1_ESM.pdf]

Supplementary Materials for "Targeted Single Primer Enrichment Sequencing with Single End Duplex-UMI"

Quan Peng Chang Xu Daniel Kim Marcus Lewis John DiCarlo
Yexun Wang

Calculation of the log likelihood ratio metric in variant calling

We here describe how to calculate the log likelihood ratio for variant calling. Suppose that at a specific site, after removing singleton and non-consensus UMIs, there are N_1 single-plex and N_2 duplex UMIs in total and among them, n_1 single-plex and n_2 duplex UMIs have the non-reference allele. If there is no mutation, all non-reference UMIs are caused by background errors (model M_0). Since the error rate follows a Beta distribution, n_1 follows Beta-binomial distribution and the probability $P(n_1|N_1, M_0)$ can be calculated as follows:

$$\begin{aligned}
P(n_1|N_1, M_0) &= \int_0^1 P(n_1|N_1, M_0, p)f(p)dp \qquad (1)\\
&= \int_0^1 \binom{N_1}{n_1} p^{n_1}(1-p)^{N_1-n_1}\frac{\Gamma(\alpha+\beta)}{\Gamma(\alpha)\Gamma(\beta)}p^{\alpha-1}(1-p)^{\beta-1}dp\\
&= \frac{\Gamma(N_1+1)\Gamma(\alpha+\beta)}{\Gamma(n_1+1)\Gamma(N_1-n_1+1)\Gamma(\alpha)\Gamma(\beta)}\int_0^1 p^{\alpha+n_1-1}(1-p)^{\beta+N_1-n_1-1}dp\\
&= \frac{\Gamma(N_1+1)\Gamma(\alpha+\beta)\Gamma(\alpha+n_1)\Gamma(\beta+N_1-n_1)}{\Gamma(n_1+1)\Gamma(N_1-n_1+1)\Gamma(\alpha)\Gamma(\beta)\Gamma(\alpha+\beta+N_1)}, \qquad (2)
\end{aligned}$$

where in (1), $f(p)$ is the Beta density function of the background error rate.

The probability of n_2 would ideally be calculated the same way if we can theoretically or experimentally characterize the error rate distribution of the duplex UMIs. Assuming the chemical reactions are independent on the two strands, the duplex error rate should in principal be the product of the single-plex error rates of the two strands. However, we observed much higher duplex error rates than theoretical calculations. On the other hand, limited by resource, we could not experimentally estimate the duplex error rate distributions in the same way as single-plex, because that would require extremely high UMI coverage. Therefore, we resorted to panel-wise mean error rates and assumed that n_2 follows a Binomial distribution. The probability

is given by

$$P(n_2|N_2, M_0) = \binom{N_2}{n_2} p_2^{n_2}(1-p_2)^{N_2-n_2} = \frac{\Gamma(N_2+1)}{\Gamma(n_2+1)\Gamma(N_2-n_2+1)} p_2^{n_2}(1-p_2)^{N_2-n_2}, \tag{3}$$

where p_2 is the panel-wise mean error rate and has distinct values for each type of base substitution. Combining (2) and (3), we have the joint probability of n_1 and n_2 under M_0 as follows:

$$\begin{aligned} & P(n_1, n_2|N_1, N_2, M_0) = P(n_1|N_1, M_0) \times P(n_2|N_2, M_0) \\ = & \frac{\Gamma(N_1+1)\Gamma(N_2+1)\Gamma(\alpha+\beta)\Gamma(\alpha+n_1)\Gamma(\beta+N_1-n_1)}{\Gamma(n_1+1)\Gamma(N_1-n_1+1)\Gamma(n_2+1)\Gamma(N_2-n_2+1)\Gamma(\alpha)\Gamma(\beta)\Gamma(\alpha+\beta+N_1)} p_2^{n_2}(1-p_2)^{N_2-n_2}. \end{aligned} \tag{4}$$

On the other hand, if there is a real mutation with an unknown allele frequency p (model M_1), n_1 and n_2 will follow Binomial distributions assuming that all non-reference UMIs are from the mutational DNA. The probability is

$$\begin{aligned} & P(n_1, n_2|N_1, N_2, M_1) = \int_0^1 P(n_1|N_1, M_1, p)P(n_2|N_2, M_1, p)g(p)dp \\ = & \int_0^1 \binom{N_1}{n_1} p^{n_1}(1-p)^{N_1-n_1} \binom{N_2}{n_2} p^{n_2}(1-p)^{N_2-n_2} dp \\ = & \binom{N_1}{n_1}\binom{N_2}{n_2} \int_0^1 p^{n_1+n_2}(1-p)^{N_1-n_1+N_2-n_2} dp \\ = & \frac{\Gamma(N_1+1)\Gamma(N_2+1)\Gamma(n_1+n_2+1)\Gamma(N_1+N_2-n_1-n_2+1)}{\Gamma(n_1+1)\Gamma(N_1-n_1+1)\Gamma(n_2+1)\Gamma(N_2-n_2+1)\Gamma(N_1+N_2+2)}. \end{aligned} \tag{5}$$

In this process, the unknown allele frequency is assumed to follow a uniform distribution, i.e. $g(p) = 1$. Given (4) and (5), the log likelihood ratio of M_1 versus M_0 is

$$\begin{aligned} & \log \frac{P(n_1, n_2|N_1, N_2, M_1)}{P(n_1, n_2|N_1, N_2, M_0)} \\ = & \log \frac{\Gamma(\alpha)\Gamma(\beta)\Gamma(\alpha+\beta+N_1)\Gamma(n_1+n_2+1)\Gamma(N_1+N_2-n_1-n_2+1)}{\Gamma(\alpha+\beta)\Gamma(\alpha+n_1)\Gamma(\beta+N_1-n_1)\Gamma(N_1+N_2+2)p_2^{n_2}(1-p_2)^{N_2-n_2}} \\ = & \log \frac{B(\alpha,\beta)B(n_1+n_2+1, N_1+N_2-n_1-n_2+1)}{B(\alpha+n_1, \beta+N_1-n_1)p_2^{n_2}(1-p_2)^{N_2-n_2}}, \end{aligned} \tag{6}$$

where $B(x, y) = \frac{\Gamma(x)\Gamma(y)}{\Gamma(x+y)}$ is the Beta function.

Supplementary Figures

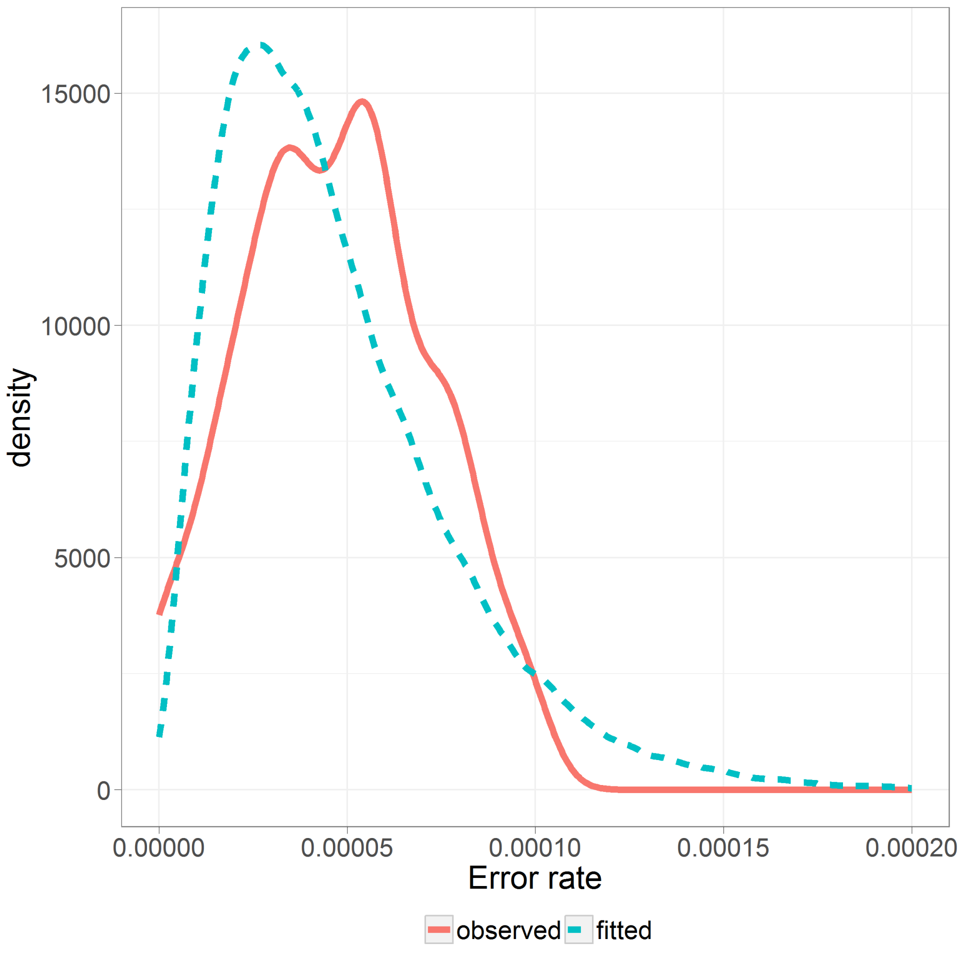

Figure S1: Observed and fitted distributions of the G-to-A background error rate based on the 10 primer panel. The fitted distribution is a Beta distribution whose parameters were estimated from the observed error rates.
